# Ecological Burden of Modern Surgery: An Analysis of Total Knee Replacement’s Life Cycle

**DOI:** 10.1016/j.artd.2023.101187

**Published:** 2023-09-18

**Authors:** Camille Delaie, Alexandre Cerlier, Jean-Noel Argenson, Jean-Charles Escudier, Raghbir Khakha, Xavier Flecher, Christophe Jacquet, Matthieu Ollivier

**Affiliations:** a Aix-Marseille Université, CNRS, ISM UMR 7287, Marseille, France; b Department of Orthopedics and Traumatology, Institute of Movement and Locomotion, St. Marguerite Hospital, Marseille, France

**Keywords:** Total knee replacement, Carbon footprint, Life cycle

## Abstract

**Background:**

It is estimated that surgical procedures account for 20%-30% of the greenhouse gases emissions from health-care systems. Total knee replacements (TKR) are one of the most frequently performed procedures in orthopaedics. The aim of this study was to identify and quantify the environmental impacts generated by TKRs, the factors that generate the most emissions, and those that can be easily modified.

**Methods:**

To calculate the life cycle carbon footprint of a posterior stabilized cemented TKR performed in a single orthopaedic surgery department, 17 TKRs performed between October 12 and 20, 2020 by 4 senior surgeons were analysed. The analysis of the life cycle included the manufacture of the implant, from raw materials to distribution; the journey made by patients and staff; and the surgery including all consumables required to facilitate the procedure.

**Results:**

The overall life cycle carbon footprint of a single TKR was 190.5 kg of CO_2_. This consisted of 53.7 kg CO_2_ (28%) for the manufacture of the prosthesis, 50.9 kg CO_2_ (27%) for travel, 57.1 kg CO_2_ (30%) for surgery, and 28.8 kg CO_2_ (15%) for waste management. This is comparable to a New York-Detroit direct flight.

**Conclusions:**

The production of a total knee prosthesis, throughout its life cycle, generates emissions with important consequences on the environment and therefore on our health. Although much data are currently missing to make precise estimates, and especially regarding benefits in terms of patient function and its impact on carbon emissions, these data serve as a starting point for other more detailed or comparative studies.

## Introduction

The negative impact of human activities on the worldwide population health is multifold. One important consideration is the effect of global warming, caused by the emission of greenhouse gases (GHGs). The latest estimates by the Intergovernmental Panel on Climate Change suggests a global temperature increase of 2°C by 2050 [1] with multiple consequences for all the ecosystems [2]. Another factor includes the effect of air pollution itself. According to the World Health Organization, it is one of the main causes of illness and premature death in the world in the 21st century [3]. In 2016, an estimated 4.6 million premature deaths were linked to outdoor air pollution, mainly as a result of cardiovascular disease (58%), chronic obstructive pulmonary disease or acute lower respiratory tract infections (18%), and lung cancer (6%). The health sector itself is a major source of air pollution and GHG emissions. In Australia, England, and the USA, the health sectors accounts for 7%, 4%, and 10% of total emissions in each country, respectively [3].

In European countries, this figure is estimated to be at 4.7% according to a report published in 2019 by *Health Care Without Harm*, although no robust study has been conducted [4]. There is still relatively little literature on the ecological impact of surgical practices; however, it is estimated that they alone account for 20%-30% of the GHG emissions of healthcare systems [5].

The surgical sector has major potential to reduce these impacts by improving practice. Although the number of studies using the life cycle assessment (LCA) method is still limited, there is a growing number of studies showing that surgical teams are increasingly interested in the emissions associated with their practice.

Total knee replacements (TKRs) are one of the most frequently performed procedures in orthopaedics [6]. With an aging Western population, the number of implanted prostheses is increasing year on year, with estimates of between 2 and 4 million TKRs being performed by the year 2050 in the USA [6]. It is a standardized procedure across the world, requiring the use of numerous implants and ancillary devices. The aim of this study was to quantify the environmental impacts generated by this type of surgery and to identify the factors that generate the most emissions and those that can be easily modified to motivate the various stake holders to work toward minimizing these impacts. The hypothesis of this study was that TKRs have a non-negligible carbon impact.

## Material and methods

This study is based on the LCA of a posterior-stabilized cemented TKR with tibial extension stem, performed by a medial parapatellar approach. The standardized LCA method (ISO 04014 and 14440) identifies and quantifies the physical flows of materials and energy associated with human activities throughout the life cycle of a product. This includes the extraction of raw materials, manufacturing, use, transport at all stages, and end-of-life treatment. It assesses the potential impacts and then interprets the results obtained as per its initial objectives. The functional unit of our study was to perform surgery using a single total knee prosthesis with a minimum of 10 years of use. The boundaries of our analysis included the following:

- Emissions generated by manufacturing
- The supply of prosthetic implants
- The manufacture of all consumables used during surgery and the management of their disposal
- The production and sterilization of surgical instruments
- The power consumption for the operation of the operating theater
- The movement of all necessary participants from their homes to the hospital.

Data on anesthesia and intraoperative pain relief, cleaning of the operating theater after each operation, treatment of liquid waste from intraoperative rinsing and suctioning, storage of patient data on a secure computer server, postoperative pain relief, stay in a conventional hospital ward, laundry service for sheets and operating theater clothes, and hospital construction were not included in this study.

### Conducting an audit

We audited all TKRs performed between 12 and 20 October 2020. A total of 17 TKRs were performed by 4 senior surgeons. We collected data and calculated average values for a functional unit allowing us to estimate the amount of potential infectious medical waste (PIMW) and noninfectious medical waste (NIMW) generated by each surgery. This included the quantity of each single-use consumable used, the duration of the operation, the time spent by the patient in the theater complex as well as the distance in kilometers between the patient's main residence and the hospital.

The brand and type of implant of the prosthesis remained consistent for each procedure. For the purposes of calculations, we used the median size for each implant.

An anonymous questionnaire was distributed to the theater team involved with patient’s care. Each member listed their professional role, the mode of transport and distance traveled between their home and hospital. This allowed us to estimate the impact of a round trip for one person in each professional category.

### Canvassing and research

Data on the composition, place of manufacture, and type of freight used for the various consumables were obtained by contacting the medical equipment suppliers concerned. After an initial contact by email, direct telephone contact was often necessary to obtain details. Information regarding the raw materials and manufacturing processes for prosthetic implants was provided by an industrial collaborator manufacturing TKR implants. The information provided was very general due to confidentiality clauses. The origin of the raw materials was estimated using data from France Stratégie published in 2020 [7]. All data concerning the sterilization of equipment was provided by the pharmacist in charge of the central sterilization department of the institution. In the absence of a sectorized meter reading, the annual electricity consumption of the operating theater was estimated using the EnergiePlus tool, which calculates the energy consumption of buildings in the tertiary sector. Finally, we recorded the weight of all consumable components, all packaging, and each prosthetic implant by weighing them on pharmaceutical scales, accurate to one hundredth of a gram.

### Estimating distances

All short- and medium-haul trips were considered to be by truck and heavy-goods vehicles. The distances in kilometers were estimated using Google Maps. For long-haul freight, we assumed that it was carried out by sea freight carriers. The distances between the respective countries were provided by a merchant navy officer.

### Calculation of LCA impact

The final total of the different environmental impacts was calculated using the Ecodesign Studio software (Altermaker Rosière, France) using Ecoinvent database [8]. We transferred all input values of our inventory for the raw materials, processes, and transport (the software used performs the calculations taking into account the weight of the product transported, in order to adapt the results to the total theoretical weight that can be transported on the same route depending on the type of freight chosen) into this database, allowing us to obtain an estimate of the life cycle carbon footprint of one single TKR.

All life cycle stages considered in our calculation were then grouped into the following 4 categories:

- The manufacture of the knee prosthesis itself, from raw materials to distribution
- The journey made by patients and staff
- The surgical procedure, including all that was necessary for its completion apart from the prosthesis
- Waste management.

These 4 categories were further divided into subcategories for a more precise description of the results.

## Results

Following data entry into the Ecodesign Studio Life Cycle Assessment software (Altermaker, Rosière, France), the life cycle carbon footprint of a single TKR procedure was 190.5 kg CO_2_ equivalent. This value corresponds to the global warming impact of a single operation.

**Figure 1:**
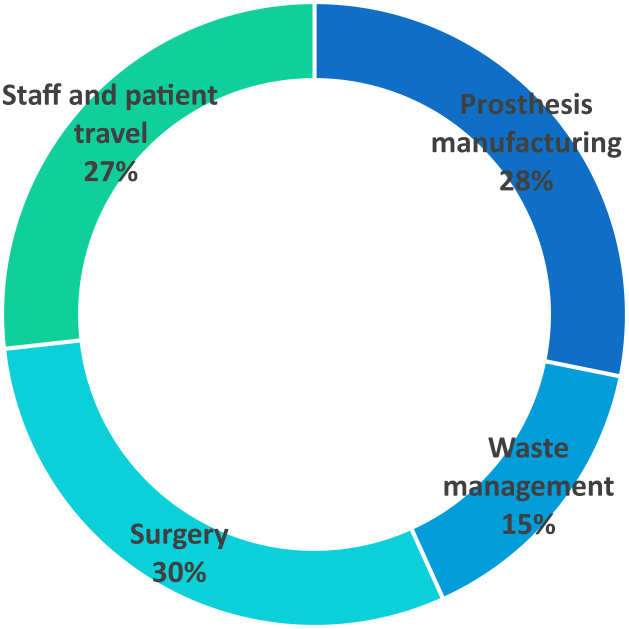
Carbon footprint by category (in percentages).

The Figure 1 shows the breakdown of the carbon footprint by category, with 53.7 kg CO_2_ (28%) for the manufacture of the prosthesis, 50.9 kg CO_2_ (27%) for travel, 57.1 kg CO_2_ (30%) for surgery, and 28.8 kg CO_2_ (15%) for waste management.

### Manufacture of the prostheses (53.7 kg CO_2_; 28%)

**Figure 2:**
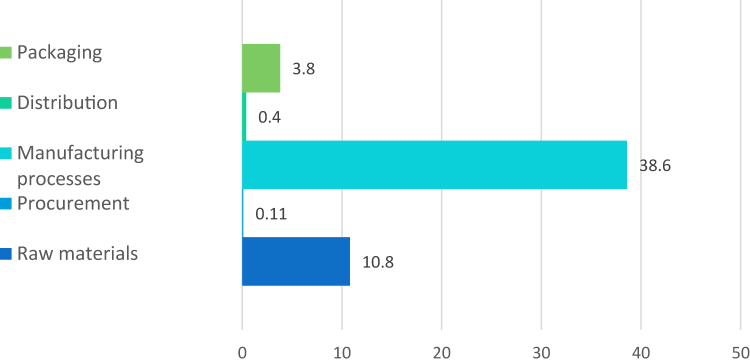
Climate impact in kg CO_2_ of the different flows in the prosthesis manufacturing category.

The extraction and manufacture of the raw materials required for the prosthetic implants represented 10.8 kg of CO_2_, with 5.8 kg CO_2_ for cobalt, 2.7 kg CO_2_ for titanium, 2 kg CO_2_ for chromium, and 0.25 kg CO_2_ for molybdenum (20.2%). For the supply of these materials, it was 0.11 kg CO_2._ The manufacturing processes accounts for a total of 38.6 kg CO_2_ for all 5 implants described (72.1%). Finally, the distribution of the prostheses from the United States to the hospital totalized 0.4 kg CO_2_ (0.7%), and 3.8 kg CO_2_ for the packaging of all the implants, including raw materials and manufacturing processes (Fig. 2).

### Travel (50.9 kg CO_2_; 27%)

An average of 31.5 kg CO_2_ was found for the patient round trip to the hospital for surgery.

For medical and associated healthcare staff, the influence of their travel for a single surgery was calculated to be 19.4 kg of CO_2._

### Surgery (57.1 kg CO_2_; 30%)

Consumables alone accounted for 53.7 kg CO_2_ or 94% of the carbon footprint for the surgery category. It also represented 28% of the total impact of the LCA, with 40.6 kg CO_2_ for raw materials, 9.3 kg CO_2_ for manufacture, and 3.8 kg CO_2_ for transportation to supply and distribute.

The carbon weight for the manufacture of instruments, ancillary equipment, and transport boxes was 0.6 kg CO_2_. Once sterilization and the return journey had been considered, there was a further 2.4 kg CO_2_ with a total of 3 kg CO_2_ for the whole process, which represented 5.3% of the surgery category.

Finally, the electricity consumption of the operating theater for the duration of an operation and monitoring in the recovery room generated the equivalent of 0.4 kg CO_2_ or 0.7% of the surgical category.

### Waste management (28.8 kg CO_2_; 15%)

The management of consumables represented 94.7% of the category with 27 kg CO_2_ emitted, compared to only 1.5 kg CO_2_ (5.3%) for waste from prosthetic implant packaging.

By separating the PIMW and NIMW, approximately 25 kg of CO_2_ was obtained for the PIMW, that is, 87% of the total, against 3.5 kg for the NIMW, representing 13% of the carbon footprint of the total waste treated.

### Other impacts

Other ecological indicators are pre-set by the software itself and were not modifiable.

**Figure 3:**
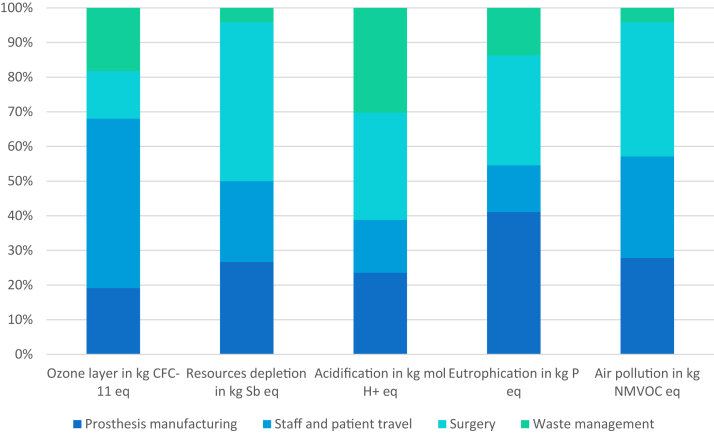
Summary of other environmental impacts in percentages by life cycle category.

The results for the other 5 impacts studied by the Ecodesign Studio software, classified by category, are summarized in the Table 1 and Figure 3. While human travel accounts for 49% of the impact on ozone depletion, the production and transport of consumables accounts for about 44% of the total impact on resource depletion. The categories of prosthesis manufacture, human travel, and surgical performance share the influence on air pollution, with 28%, 29%, and 39% of the total for this impact, respectively.

**Table 1:** Results for other environmental impacts by life cycle category.

Categories	Ozone layer in kg CFC-11 eq	Resources depletion in kg Sb eq	Acidification in kg mol H+ eq	Eutrophication in kg P eq	Air pollution in kg NMVOC eq
Prosthesis manufacturing					
Raw materials	9.92E-07	8.86E-02	9.76E-02	6.32E-03	3.96E-02
Manufacturing processes	1.69E-06	2.74E-01	1.85E-01	1.53E-02	1.07E-01
Transports	8.27E-08	3.35E-04	8.65E-03	3.43E-05	4.68E-03
Packaging	1.68E-07	3.33E-02	1.79E-02	1.26E-03	1.35E-02
Total	2.94E-06	3.96E-01	3.09E-01	2.29E-02	1.65E-01
Travels					
Patient	4.71E-06	2.17E-01	1.24E-01	4.67E-03	1.07E-01
Hospital staff	2.80E-06	1.30E-01	7.67E-02	2.85E-03	6.75E-02
Total	7.51E-06	3.47E-01	2.00E-01	7.52E-03	1.74E-01
Surgery					
Consumables	1.80E-06	6.58E-01	3.62E-01	1.59E-02	2.11E-01
Instruments et ancillaries	2.75E-07	2.03E-02	4.05E-02	1.58E-03	1.63E-02
E consumption of OR	3.63E-08	2.76E-03	3.24E-03	2.50E-04	2.70E-03
Total	2.11E-06	6.81E-01	4.06E-01	1.77E-02	2.30E-01
Waste management					
Consumables	2.65E-06	5.84E-02	3.75E-02	7.23E-03	2.33E-02
Packaging	1.50E-07	3.30E-03	2.12E-03	4.08E-04	1.32E-03
Total	2.80E-06	6.17E-02	3.96E-02	7.64E-03	2.46E-02

## Discussion

The main finding of the present study is that undertaking TKR surgery generates emissions on our planet and health which are not inconsiderable. For a single operation, the CO_2_ emissions generated 190.5 kg CO_2_ eq which is a little more than a New York–to–Detroit direct flight.

When we extrapolate this figure to the 700 TKRs performed in our department and then to the 500 000 implanted in the United States in 2020 [6], we obtain a GHG balance of 95,000 tCOeq_2_. This represents the equivalent burden of the traffic of 20.470 cars for 1 year and would require the growth of 1.5 million trees for 10 years to capture all this carbon. However, one of the most important biases of this descriptive study is the lack of analysis of the benefit in terms of patient function and the impact of this benefit on TKR carbon footprint. Indeed, by switching from driving to walking or cycling for one trip per week, a person's carbon emissions can be reduced by about 0.5 tons per year. Therefore, TKR can potentially provide a significant carbon footprint benefit for a relatively low carbon cost.

The subcategory of manufacturing and distribution of consumables has the largest carbon footprint, accounting for 28% of total CO_2_ emissions. These results are lower than those found in the English cataract study by Morris with 53% of the total impact. However, it should be noted that they also included the supply of medicines and food in this category, which was not considered in our study [9].

The presence of a large quantity of plastic and cotton by-products is largely responsible for the fact that consumables are at the top of the emissions lists. With all categories of impact combined, there is a more marked effect on resource depletion and ocean acidification.

These data are similar to those found in the American study by Campion and Thiel [10], which compared environmental consequences of giving birth by vaginal delivery or caesarean section.

In our study, 38.6 kg of CO_2_ is emitted from the overall manufacturing process of the implants. This represents a significant percentage in all the impact categories studied. This figure can be accounted for by the fact that there is a need to heat the various metals to very high temperatures as well as transportation from the United States. There is therefore a high consumption of fossil fuels. In addition, the accumulation of numerous specialized processes to achieve the final implants quickly increases these figures. Due to the confidential nature of the specific steps taken to manufacture the implant designs, these figures are likely to represent a significant underestimation of the overall figure.

The extraction and transport of raw materials, such as titanium or cobalt, do not seem to have a major impact on the overall LCA. We can account for this when considering the low weight of a prosthesis and therefore the very small amount of each raw material needed.

When considering the impact of transportation, the patient’s journey to the hospital alone has a significant potential impact on climate change and the depletion of the ozone layer. This contribution is undoubtedly underestimated since we did not take into consideration the return trips made during preoperative and postoperative consultations or the preoperative anesthesia consultation. Nevertheless, it is clear that patients travel a greater distance than staff to the hospital, with an average of 133 km round trip for patients vs 24.5 km round trip for hospital staff. Whereas hospital staff live relatively close to the hospital, the long distance traveled by patients is explained by the fact that our department is a regional referral center for prosthetic surgery, with a fairly large population base.

It is also worth noting that all the transport associated with the movement of raw materials and the distribution of prosthetic implants or consumables is not represented to any great extent in the overall impacts, despite the long distances covered. Their calculation is defined by the weight of the product in relation to the total weight transported by the means of transport studied. The total weight of approximately 12 kg, including the prostheses, therefore, is relatively small in comparison to the flows of goods that pass through the world.

Furthermore, our assumption that all long-distance transport is carried out by sea freighters may be wrong. This type of journey often takes approximately 10 days, whereas the supply of medical equipment may sometimes require urgent transfers of products, which are then carried by cargo planes. We have simulated calculations that replace sea journeys with air journeys, which demonstrates an increase in all the impacts studied, significant for ozone depletion and climate change (231 kg CO_2_ eq or +21%).

Finally, waste management has a major impact on global warming and ozone depletion. It is almost exclusively carried out by incineration, which emits a large quantity of polluting substances (gases and particles) into the air. As expected, the management of hazardous waste has the greatest impact, 3 times higher than that of household waste.

This study has some limitations. A significant part of our study is based on assumptions and approximations. The difficulties encountered with precise manufacturing processes for medical equipment from medical companies meant certain assumptions had to be made. This is likely to have resulted in missing or incomplete data. We tried to compensate for this with the hypotheses that seemed most plausible or those that seemed to underestimate rather than overestimate the overall impact. Other important factors were excluded from the outset, including the impact of anesthesia during surgery. It is now clearly established that some anesthetic gases have an impact on global warming [11] desflurane in particular, which generates emissions equivalent to 2540 times those of CO_2_ [12].

Although some teams are limiting its use in favor of other less polluting gases, it is still widely used. Nevertheless, as only one general anesthesia and 16 spinal anesthetics were performed during the period of our audit, we assumed that its overall impact would be limited.

Other data, such as the construction of the hospital or the patient's stay in hospital, were not taken into account, again leading to an underestimation of the overall impact.

The price of full access to the Ecoinvent database and Ecodesign Studio software license was a barrier to the study. Free registration on the Ecoinvent website gave access to the list of materials, processes, and transport listed in the database but did not give direct access to the input flows recorded and the output flows obtained. Free access to all the data in the “IMPACTS database” enabled us to make a more accurate calculation.

However, this study represents one or the rare analysis of the ecological impact of modern surgery, and one of the first concerning orthopaedic surgery.

How can we improve?

Between 23%-30% of the waste produced by a hospital comes from the operating theater [5]. Since a large proportion is represented by plastics and cardboard, by performing selective sorting in dedicated bins in each operating theater, for recycling nonhazardous waste could help to reduce the impact. An American study published in 2012, after an audit of the waste produced by 10 total hip replacements and 10 TKRs, found that 20% of the total waste was recyclable [13]. A comprehensive study by Thiel et al [14] on strategies to reduce GHG emissions from laparoscopy, found that recycling has little impact (<5%) if implemented in isolation. However, it is the simplest action to implement on a daily basis. It is also estimated that 50%-85% of the waste considered as PIMW does not belong there. This led to higher costs and a higher environmental impact [15]. If sorting recommendations for PIMW were to exist, such rules would need to be adopted at national level to encourage proper implementation by all healthcare staff. Our projections in the LCA software show a 7.4% reduction in the impact on global warming with optimized PIMW sorting. Other considerations include removing the process of package leaflets for implant box identification and making this a digital process.

In the twin aims of reducing our waste and our use of plastics, action on how we use consumables can be of paramount importance. From the 1980s onwards, concerns about blood-borne diseases encouraged the development of single-use equipment.

There are conflicting studies on the benefits of reusable vs single-use on the ecological footprint [16,17]. Several authors suggest that a return to reusable and resterilizable drapes and swabs are some of the key elements to reducing the impact in the operating theater with a reduction in CO_2_ emissions of 4% according to Thiel's study [14].

One could also imagine the use of recycled plastic for the manufacture of packaging or even of all single-use material, as well as organic cotton for all compresses.

In reducing consumables, and therefore waste, there is likely to be a positive impact on reduction on the environmental impact and costs since waste corresponds to 20% of health expenditure [18]. The packaging containing the equipment should be personalized to adapt them to each practice or block and thus avoid wasting unused equipment.

Before the surgical procedure, the patient is required to make multiple return trips to the hospital for all pre- and post-operative consultations. While clinical examination remains indispensable during some of these consultations, others can be envisaged through teleconsultation. Several dedicated websites and applications have been set up, particularly since the coronavirus disease 2019 pandemic.

It is difficult to envisage the complete manufacturing process of TKRs to take place in any European or American country, as not all raw materials are readily available. However, countries with highly specialized industries, carrying out all the manufacturing processes (forge, foundry, machining, and so on) might be achievable and thus limit the multiple round trips around the world.

The research and development sector must invest in more virtuous innovations, whether in the field of consumables, prosthetic manufacturing, or the manufacture of all the machines present in the surgical rooms.

Finally, even if, the power consumption of the operating theater has a limited influence in this study, the necessary transformations of the operating theaters should be carried out to improve energy efficiency. Reducing the overall consumption when the rooms are not occupied by using presence sensors, installing light-emitting diode bulbs, or even using natural light through windows are part of the strategy that may be implemented.

The transition to electricity generation, via renewable energy, will help to make the change.

All these proposals are part of the 5Rs strategy: Reuse, Reduce, Recycle, Rethink, and Research.

## Conclusions

The production of a total knee prostheses, throughout its life cycle, generates emissions with important consequences for the environment and therefore impact our health. Although much data are currently missing to make precise estimates, and especially benefits in terms of patient function and its impact on carbon emissions, these data serve as a starting point for other more detailed or comparative studies.

## Funding

The authors did not receive support from any organization for the submitted work.

No funding was received to assist with the preparation of this manuscript.

No funding was received for conducting this study.

No funds, grants, or other support was received.

## Conflicts of interest

Jean-Noel Argenson is a paid consultant for Zimmer Biomet; is a part of editorial board for the Journal Bone Joint Surgery; and is a board member for the SOFCOT and ESSKA. Matthieu Ollivier is a board member for the SOFCOT and ESSKA; is a part of editorial board for the Knee Sports Traumatology and Arthroscopy; and is a paid consultant for STRYKER and NEWCLIP. Xavier Flecher is a paid consultant for Zimmer Biomet and is a board member for the SOFCOT and ESSKA. All other authors declare no potential conflicts of interest.

For full disclosure statements refer to https://doi.org/10.1016/j.artd.2023.101187.

## Author contributions statement

MO, AC, CJ, JNA, XF designed the protocol. CD, JCE, RK performed database analysis. CD, AC, JCE wrote the initial draft. CD, AC, JNA, JCE, RK, XF, CJ, MO edited the different version of the draft. CD, AC, JNA, JCE, RK, XF, CJ, MO approved of the submitted and final versions.

## Ethics approval

Not applicable.

## Consent for publication

Not applicable.

## Availability of data and material

Not applicable.

## Appendix A Supplementary data

Conflict of Interest Statement for Cerlier

Conflict of Interest Statement for Delaie

Conflict of Interest Statement for Jacquet

Conflict of Interest Statement for Escudier

Conflict of Interest Statement for Argenson

Conflict of Interest Statement for Ollivier

Conflict of Interest Statement for Flecher

Conflict of Interest Statement for Khakha
