# Supplementary material for: Ecological Burden of Modern Surgery: An Analysis of Total Knee Replacement’s Life Cycle
Source: Arthroplast Today. 2023 Sep 18;23:101187. doi: 10.1016/j.artd.2023.101187 (PMC10514426; doi:10.1016/j.artd.2023.101187)
Supplement: Conflict of Interest Statement for Argenson [file mmc5.docx]

# INDIVIDUAL CONFLICT OF INTEREST STATEMENT

***American Association of Hip and Knee Surgeons***

(Adopted from the American Academy of Orthopaedic Surgeons disclosure statement)

The following form **must be filled out completely and submitted by each author (example, 6 authors, 6 forms).**

**All items require a response. If there is no relevant disclosure for a given item, enter "*None*.”**

**Manuscript Title**

**Ecological burden of modern surgery: an analysis of Total Knee Replacement’s life cycle.**

1. Royalties from a company or supplier (The following conflicts were disclosed)

NO

2. Speakers bureau/paid presentations for a company or supplier (The following conflicts were disclosed)

NO

3A. Paid employee for a company or supplier (The following conflicts were disclosed)

NO

3B. Paid consultant for a company or supplier (The following conflicts were disclosed)

YES Consultancy for ZIMMER BIOMET

3C. Unpaid consultants for a company or supplier (The following conflicts were disclosed)

NO

4. Stock or stock options in a company or supplier (The following conflicts were disclosed)

NO

5. Research support from a company or supplier as a Principal Investigator (The following conflicts were disclosed)

NO

6. Other financial or material support from a company or supplier (The following conflicts were disclosed)

NO

7. Royalties, financial or material support from publishers (The following conflicts were disclosed)

NO

8. Medical/Orthopaedic publications editorial/governing board (The following conflicts were disclosed)

YES for the Journal Bone Joint Surgery (editorial board)

9. Board member/committee appointments for a society (The following conflicts were disclosed)

YES for the SOFCOT and ESSKA

**Each author must sign AND print or type his/her name, date and submit a separate form**

In addition, one BLINDED Conflict of Interest form (no author names used) should be submitted per manuscript with all author disclosures.

Jean-Noel Argenson
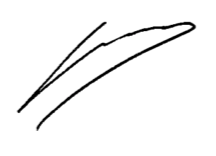
 13/01/22

Author Name (Print or Type) Author Signature Date
